# Health Consumer Engagement, Enablement, and Empowerment in Smartphone-Enabled Home-Based Diagnostic Testing for Viral Infections: Mixed Methods Study

**DOI:** 10.2196/34685

**Published:** 2022-06-30

**Authors:** Cynthia LeRouge, Polina Durneva, Victoria Lyon, Matthew Thompson

**Affiliations:** 1 Department of Information Systems and Business Analytics College of Business Florida International University Miami, FL United States; 2 Primary Care Innovation Lab Department of Family Medicine University of Washington Seattle, WA United States; 3 Get-Grin Inc Austin, TX United States

**Keywords:** smart HT, mHealth, patient engagement, patient enablement, patient empowerment, diagnostic testing, viral infection, patient activation, consumer health informatics, influenza, home testing, mobile phone

## Abstract

**Background:**

Health consumers are increasingly taking a more substantial role in decision-making and self-care regarding their health. A range of digital technologies is available for laypeople to find, share, and generate health-related information that supports their health care processes. There is also innovation and interest in home testing enabled by smartphone technology (smartphone-supported home testing [smart HT]). However, few studies have focused on the process from initial engagement to acting on the test results, which involves multiple decisions.

**Objective:**

This study aimed to identify and model the key factors leading to health consumers’ engagement and enablement associated with smart HT. We also explored multiple levels of health care choices resulting from health consumer empowerment and activation from smart HT use. Understanding the factors and choices associated with engagement, enablement, empowerment, and activation helps both research and practice to support the intended and optimal use of smart HT.

**Methods:**

This study reports the findings from 2 phases of a more extensive pilot study of smart HT for viral infection. In these 2 phases, we used mixed methods (semistructured interviews and surveys) to shed light on the situated complexities of health consumers making autonomous decisions to engage with, perform, and act on smart HT, supporting the diagnostic aspects of their health care. Interview (n=31) and survey (n=282) participants underwent smart HT testing for influenza in earlier pilot phases. The survey also extended the viral infection context to include questions related to potential smart HT use for SARS-CoV-2 diagnosis.

**Results:**

Our resulting model revealed the smart HT engagement and enablement factors, as well as choices resulting from empowerment and activation. The model included factors leading to engagement, specifically various intrinsic and extrinsic influences. Moreover, the model included various enablement factors, including the quality of smart HT and the personal capacity to perform smart HT. The model also explores various choices resulting from empowerment and activation from the perspectives of various stakeholders (public vs private) and concerning different levels of impact (personal vs distant).

**Conclusions:**

The findings provide insight into the nuanced and complex ways health consumers make decisions to engage with and perform smart HT and how they may react to positive results in terms of public-private and personal-distant dimensions. Moreover, the study illuminates the role that providers and smart HT sources can play to better support digitally engaged health consumers in the smart HT decision process.

## Introduction

### Emerging Smartphone-Enabled Home Testing

“If we can get a test that everyone wakes up and, just like they put in their contact lenses, they take a test, and if it turns positive, they stay at home...it will stop the vast majority of transmission and cause these outbreaks to disappear in a matter of weeks” [1].

Health consumers are increasingly taking a more substantial role in decision-making and self-care of their health [2,3]. This role includes using home-based diagnostic tests (also called home tests, self-tests, or home-use tests), where new technologies can expand and enhance our ability to “examine the body’s inner workings and preoffer an exact explanation of the person’s present medical condition” [4]. Home testing is convenient and enhances the efficiency of obtaining test results. Home tests are generally publicly available (eg, can be sold over the counter). They allow health consumers to obtain and test self-collected specimens from their location. Home test consumers can interpret test results independently without the help of trained health professionals [5]. Home tests differ from home collection kits (eg, 23AndMe), which require individuals to self-collect samples at home, mail them to a laboratory or clinic for analysis, and obtain the results via phone or a web-based portal. The more immediate results of home testing also potentially help to avoid the spread of infections [6]. Currently, numerous biotechnical institutions are targeting new frontiers in self-diagnostic innovations for viral infections that aim to be client centered, technically robust, and financially affordable [7-9].

Health information technology (HIT) is now seen as a fundamental aspect of patient care as it stimulates patient engagement and encourages personal health management [10]. Furthermore, health care providers increasingly demand patient interaction with digital health technologies to enroll in care, access personal health information, communicate with providers, and monitor health [11]. Coupling technology with testing supplies needed to obtain specimens (eg, tubes, containers and swabs) for home tests can support and reinforce the decision process and ultimate health care path resulting from diagnostic testing. Specifically, smartphone-supported home testing (smart HT) is receiving increasing interest and can give health consumers the ability to play a more active role in the testing experience [12-14].

Smart HT content and features support engaged health consumers in testing safely and independently in their homes, learning how to manage their illness based on test results, learning how to manage the spread, and sharing test results for personal or public health networks electronically [15,16]. Smart HT may be particularly promising to support personal and public health concerns (ie, contribute to public health surveillance and management) related to respiratory viruses, such as influenza and COVID-19. Furthermore, smart HT may leverage new convenient means of connecting to care options, potentially minimizing the spread of respiratory viruses. Specifically, a smart HT accommodating a telemedicine encounter allows enabled health consumers to act on results through an at-home connection with health providers, thereby expediting suitable personal care and minimizing contact with others when quarantine is appropriate.

Consumer health tools, including smart HT, must be effectively designed and used [10]. Therefore, it is increasingly important to understand consumer HIT patterns, including who uses specific technologies, how technologies are accessed, factors associated with their use, and perceived and actual benefits [10]. Regarding the practicalities of home testing success, there is an underlying assumption that the home-based tester is engaged in the testing process, enabled to perform the test, and empowered to act in ways conducive to their health (and the health of others) after receiving results. These assumptions involve multiple critical decisions that health consumers must make regarding acquiring the test, self-performing the diagnostic test, and choosing healthy choices and behaviors after testing (particularly in response to positive test results).

### Smart HT Empowerment and Activation Journey

For infectious disease management, the goal of using smart HT is for individuals to receive test results and take the best course of action based on their test results for themselves and society. A holistic understanding of this journey is required for smart HT to positively affect both individual and public health. Indeed, feasibility cannot be genuinely achieved until health consumers intending to use smart HT are aware, engaged, and empowered and ultimately respond actively to the test results.

**Figure 1:**
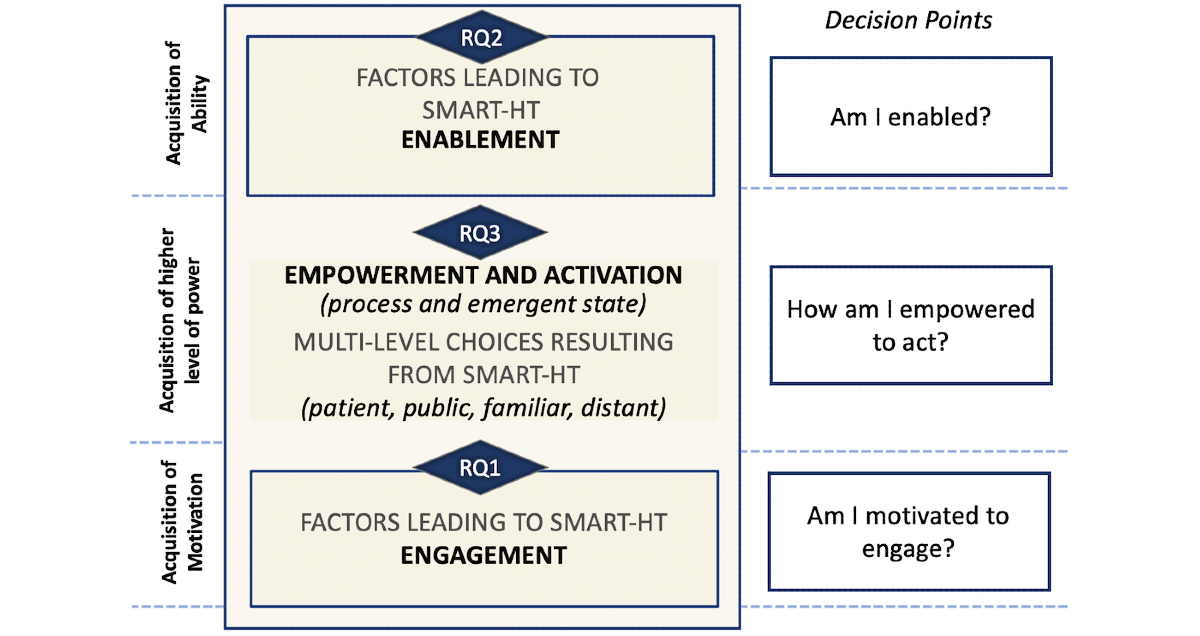
Smartphone-supported home testing (Smart HT)–Empowered Activation Model (research questions, RQ's, highlighted).

Figure 1 illustrates a patient engagement, enablement, empowerment, and activation process model (hereafter referred to as the Smart HT–Empowered Activation Model) informed by work, resulting from an extensive literature review of these states by Fumagalli et al [17]. This process model was adapted to the context of smart HT. The path to empowered activation includes healthy consumers’ responses to critical personal assessments, leading to emergent states of engagement, enablement, and empowerment. Our model shows that achieving each state is ultimately based on a series of autonomous assessments in response to the following types of questions:

- Am I motivated to engage with the test? (state of engagement)
- Am I enabled to perform the test? (state of enablement)
- How am I empowered to act on the results of the test? (state of empowerment and activation)

It is important to note that this process model assumes that a health consumer is aware of and has access to smart HT. Awareness of smart HT can result from the potential user being the recipient of marketing or trial recruitment efforts (eg, through trial enrollment, marketplace, and provider) that promote the acquisition of smart HT. The factors associated with awareness have been addressed in prior research [14,18]. Health consumers who are aware of and have access to smart HT can become engaged, enabled, empowered, and activated through smart HT use (as illustrated in Figure 1). Descriptions of these emergent states (engagement, enablement, empowerment, and activation) are presented in Textbox 1.

**Descriptions of engagement, enablement, empowerment, and activation.**

**Engagement**

- Engagement refers to individual motivation to participate in self-management behaviors.

**Enablement**

- Enablement comprises 2 components:
  having appropriate knowledge, skills, and abilities to understand one’s health condition and make decisions.
  having appropriate contexts to learn such knowledge, skills, and abilities

**Empowerment and activation**

- Empowerment is a consequence of enablement and engagement and takes a form of an emergent state and process:
  As an emergent state, empowerment allows individuals to have an active role in their own care.
  As a process, empowerment is a process of “activating” individuals, indicating that someone gains knowledge of how to manage their health condition and access appropriate health care.

Note: Descriptions derived from a literature review performed in Fumagalli et al [17].

According to Fumagalli et al [17], engagement and enablement are critical for achieving empowerment and activation. In the context of smart HT, engaged health consumers are those who develop the motivation to engage with smart HT, specifically to test for their health condition by using smart HT. However, engagement alone is not sufficient to achieve empowerment and activation, as it is also essential for health consumers to become enabled. Enabled health consumers have the appropriate knowledge of how to complete smart HT and the capacity to perform the test. In the context of smart HT, the technological aspect of the test is an important component supporting health consumers’ efforts to complete the test successfully. Therefore, various characteristics of technology need to be considered when exploring enablement.

As health consumers acquire engagement and enablement, they achieve an emergent state of empowerment and activation. When viewed as an emergent state, empowered health consumers possess a higher level of power and appreciation for their role in the health care process [17]. An activated patient is “someone who has...the skills and behavioral repertoire to manage their condition, collaborate with their health providers, maintain their health functioning, and access appropriate and high-quality care” [17]. Empowerment and activation can be a recursive process for smart HT as initial empowerment may be fueled by individual steps completed successfully as smart HT testing is enacted, which further fuels empowerment for downstream steps.

When empowerment is coupled with activation, possession of knowledge, skills, attitudes, and self-awareness can improve individuals’ life situations. In the context of smart HT, health consumers become empowered to enact behaviors that could affect them personally (eg, self-care) and affect the public (eg, self-isolation to prevent spread) upon receiving positive test results.

The attainment of enablement and engagement is affected by multiple factors. The extensive literature that informed the model in Figure 1 provides some insight into the basic concepts and definitions of engagement, enablement, empowerment, and activation (Textbox 1) [17]. However, we still have a limited understanding of the factors affecting health consumers’ path toward engagement and enablement and health consumer choices resulting from empowerment and activation.

HIT studies that address the antecedents of consumer health technology use [19-21] do not generally distinguish the factors related to moving toward states of engagement and enablement. Instead, these HIT studies tend to focus on demographic factors (eg, race, sex, and socioeconomic status), health conditions (eg, overweight or obese), or adherence to healthy behaviors (eg, eating or physical activity patterns) holistically affecting adoption without recognizing the emergent states in the process leading to use or acceptance [21-25]. Furthermore, the literature on HIT adoption does not explore the various pathways for smart HT–empowered action. Therefore, in the case of smart HT, we know little about consumers’ choice of options and intentions once enabled by the test results. Thus, to fully uncover and understand consumer patterns, it is imperative to understand the factors influencing the enablement and engagement states and the movement to the empowerment and activation process and emergent states.

This study aimed to gain a deeper understanding of the process related to achieving patient activation for smart HT by understanding the factors that affect decisions to move along the empowered activation process. We addressed this exploration in the context of respiratory viral infection (RVI), which is a serious public health threat [26], meriting smart HT exploration and consideration. We specifically targeted the 3 decision points, particularly interactions with smart HT, by addressing the following research questions (RQs):

- RQ1: For a health consumer aware of smart HT for RVIs, what factors lead to the emergent state of engagement with smart HT?
- RQ2: For a health consumer aware of smart HT for RVIs, what factors lead to the emergent state of enablement to perform smart HT?
- RQ3: For a health consumer who is enabled and engaged with smart HT for RVIs, what choices result from the emergent state of empowerment to act upon the results (particularly positive test results) obtained through smart HT?

## Methods

### Study Overview

The focus of this project is a pilot study of an innovation called flu@home, a smart HT for influenza. This flu@home pilot is part of a more extensive research study called the Seattle Flu Study, which explored the feasibility of using home-based testing for the surveillance and public health management of viral outbreaks [27,28]. The flu@home smart HT contains 2 major components: a mobile app and an influenza test kit. The mobile app was designed to screen participants experiencing influenza-like illness (ILI) symptoms and facilitate testing of participants. Once screened, the participants used the app to consent to the research protocol and order their influenza test kit. The influenza test kit included materials adapted from an existing point-of-care lateral flow test called the QuickVue Influenza A+B test (Quidel Corporation). Once participants received the influenza test kit, the mobile app gave them instructions to complete the self-test.

**Figure 2:**
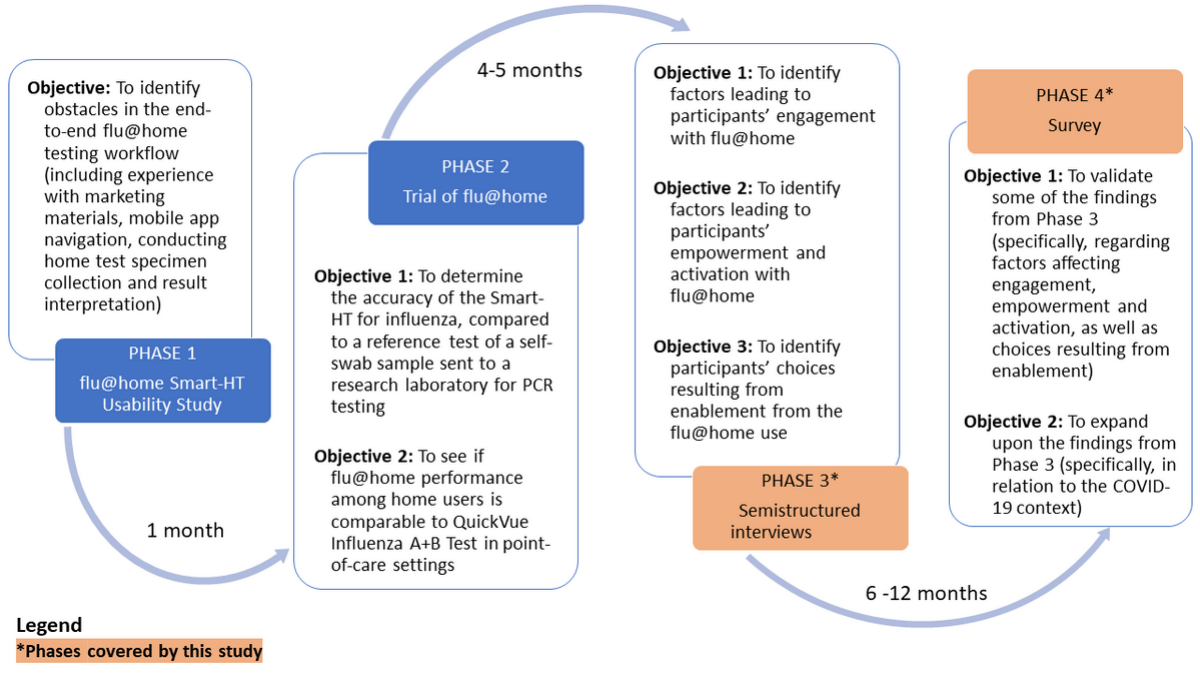
Study phases (phases 3 and 4 [shown in orange] are the objectives that relate to the scope of this paper). PCR: Polymerase chain reaction.

The flu@home pilot comprised four phases: (1) flu@home smart HT usability study, (2) trial of flu@home, (3) semistructured interviews regarding the experience of using flu@home, and (4) a survey of those who used flu@home. Figure 2 summarizes the 4 phases of the study and describes the objectives of each phase.

Phase 1 (the flu@home smart HT usability study) focused on the development of flu@home to meet usability standards. Participants from phase 1 usability assessments used to inform the software development were not recruited for the subsequent phases.

After the development of flu@home, phase 2 (trial of flu@home) was conducted to determine its accuracy. During phase 2, the participants had a chance to experience the actual flu@home test. Phase 2 participants were also recruited for phases 3 and 4, which explored participants’ experiences with the flu@home test, various factors affecting engagement and enablement, and choices resulting from empowerment and activation.

These multiple phases of the flu@home pilot leveraged mixed methods (both qualitative and quantitative). Mixed methods can be valuable for developing and evaluating complex interventions such as smart HT [29,30]. Studies have recognized that mixed methods add value by identifying the mechanisms of complex problems, increasing the validity of the findings, and providing a deeper understanding of the phenomenon of interest [31,32]. In this study, we used an exploratory sequential design described by Creswell and Clark [33], which was used first to explore a phenomenon of interest (through qualitative methods) and then clarify the findings by leveraging quantitative methods. In line with this approach, we collected qualitative data (phase 3) to explore the decision points and factors associated with engagement, enablement, and empowerment. We then conducted a quantitative phase (phase 4) to validate and further explore various factors affecting decision points associated with engagement and enablement and choices resulting from empowerment and activation*.*

The inclusion criteria for phase 2, which also applied to phases 3 and 4, involved eligible participants who were aged ≥18 years, spoke English, had an iPhone or iPad, and had an ILI (defined as the presence of a cough and at least one or more of the following symptoms: fever, chills or sweats, muscle, body aches, or feeling tired or more tired than usual). Recruitment was limited to individuals in the lower 48 states of the United States to ensure that they received their flu@home test kit within 2 days of enrolling in the study. Overall, 97.9% (724/739) of participants who completed phase 2 consented to be contacted for future, related research and were eligible to participate in phases 3 and 4.

### Semistructured Interviews

Participants from phase 2 (trial of flu@home) were invited to participate in in-depth, semistructured interviews to share their experiences with the flu@home smart HT and their beliefs and attitudes toward using smart HT (for influenza) in the future. Semistructured interviews involved a series of predetermined, open-ended questions with probes and prompts to elicit further information about the phenomenon of interest [34]. We used a phenomenological approach to conduct semistructured interviews. Phenomenology allows researchers to explore human experiences to elicit meanings for individuals by analyzing their perceptions of the phenomenon of interest [35]. In particular, we leveraged hermeneutic phenomenology [36]. In hermeneutic phenomenology (as distinguished from transcendental phenomenology), pre-existing knowledge and researchers’ understanding of concepts related to the phenomenon of interest cannot be fully bracketed in interpreting participants’ descriptions of the phenomenon [37]. Phenomenological hermeneutic semistructured interviews were leveraged to gain insights into factors influencing decision points associated with engagement, empowerment, and activation, as well as choices resulting from enablement [38-41].

Our interview guide (Multimedia Appendix 1 [18,42-46]) aligns with the phenomenological interview method described by Bevan [47]. The interview guide included a series of broad and open-ended questions that allowed participants to express their opinions extensively and freely. We recognized three aspects in developing a phenomenological interview guide: (1) contextualization (understanding of participants’ context in which the experience of the phenomenon of interest is situated), (2) apprehending the phenomenon (questions related to the specific phenomenon of interest), and (3) clarifying the phenomenon (eg, imaginative variation) [47]. In alignment with the principle of contextualization, our interviews started with general questions related to participants’ general attitudes and behaviors related to health. After discussing participants’ general attitudes and behaviors related to health, our interview questions transitioned to exploring participants’ experiences with flu@home and the values and gains associated with using flu@home (ie, apprehending the phenomenon). Although not specifically referencing our high-level constructs of interest in the discussion, these questions aimed to explore engagement, enablement, and activation in more detail. Finally, we clarified the smart HT phenomenon using imaginative variation [47]. Imaginative variation is leveraged when the researcher understands a specific element of a participant’s experience, which is then applied to varying its structural components to uncover the invariant parts. We used imaginative variation to explore hypothetical situations, such as using smart HT in the future for influenza and other medical issues.

Prior literature was reviewed to inform the semistructured interview questions and a priori coding schema for data analysis. We looked to the literature that would provide more insight and detail into elements of the conceptual framework used in this study, precisely, factors affecting engagement and enablement and choices resulting from empowerment and activation (Figure 1). Two studies that included extensive literature review especially informed our interview content and a priori coding schema: the Fumagalli Concept Map of Engagement and Neighboring Concepts [17] and the Digital Health Engagement Model (DIEGO) [18].

Fumagalli et al [17] indicated that engagement manifests in patients’ behaviors to improve their role in health care. Patient motivation to engage in such behaviors can be determined by intrinsic influences (eg, proactive role in health care resulting in the patient making appointments, staying informed about treatment options, and others) and extrinsic influences (eg, specific characteristics of health intervention) [17,48]. In alignment with this view of engagement, we focused on 2 categories of factors affecting decision points associated with engagement: intrinsic and extrinsic influences.

To inform categories of factors associated with enablement, we referred to applicable high-level concepts in the DIEGO [18] The DIEGO model contains multiple categories of factors associated with an individual’s enrollment in and engagement with digital health interventions. Some categories of factors in the DIEGO model point to specific aspects of health consumers’ interactions with digital health, which can enable health consumers to complete a digital health intervention. We leveraged factors particularly pertinent to health consumers’ ability to complete the intervention: considering the quality (of the HIT) and assessing personal capacity (for using the HIT).

Finally, to inform the interview questions and high-level codes for categories of choices resulting from health consumers’ empowerment and activation, we considered the different levels of impact (*patient* and *public*). These different levels of impact were partially informed by the DIEGO, which examined individual-level and public-level engagement with digital health interventions [18]. We subdivided these categories to consider proximal associations (familiar and distant). Overall, 4 multilevel choices emerged: *patient-familiar*, *patient-distant, public-familiar,* and *public-distant. Patient-familiar* actions are defined as actions that individuals take to care for their illness in a familiar setting (eg, visiting primary care providers or self-managing the illness). *Patient-distant* actions are ways in which individuals can seek care in a more distant manner (eg, visiting urgent care or seeking telemedicine consultations). *Public-familiar* actions are actions that individuals take to prevent the spread of their illness to family, friends, coworkers, and people they interact with frequently. Finally, *public-distant* actions are those that individuals take to prevent the spread of their illness in their community at large, such as sharing their test results to contribute to the awareness of the illness in their community.

The interview sample size was guided by data saturation, which is the point at which additional data collection no longer generates any new insights [49]. Prior studies with similar study designs indicate that data saturation can generally be achieved in data samples ranging from 10 to 40 individuals [50-53]. Therefore, in alignment with prior studies and general recommendations for sample sizes, we determined a minimum of 20 interviews to be an appropriate target number.

We recruited participants in 3 waves to include a diverse representation of geographic locations and ages (to accurately reflect the targeted user population). The first wave of the selection process comprised sorting participants into age groups (18-24, 25-34, 35-44, 45-64, and ≥65 years) and randomly inviting them to interviews, selecting participants from each group. During this initial recruitment wave, we sent 60 participants study invitations assuming that 50% of participants would sign up for an interview based on completion rates of home collection studies for other health conditions [54-56]. In the 2 subsequent waves of recruitment, the proportion of participants recruited from each age group was adjusted to ensure sample representation across all age groups. Recruitment continued until at least 3 participants from each age group were interviewed in each stratum. A total of 115 participants were invited, and 31 (26.9%) completed the interviews. Table 1 summarizes the participants’ demographics in phase 2 (trial of flu@home) and phase 3 (specifically, participants who were invited to participate in the semistructured interviews and participants who completed the semistructured interviews).

Confidential 40- to 60-minute semistructured interviews were conducted using Zoom videoconferencing [57]. A total of 3 research team members with backgrounds in HIT, consumer technologies, and public health conducted the interviews. In cases where 2 research team members were present, 1 team member served as the lead interviewer. The other team members served the role of scribe and active listeners. The 2 team members conducting the interview held a debriefing session after each interview to discuss key points to consider for coding purposes and discuss the interview protocol flow. Deidentified interview transcripts were uploaded to Dedoose, a software for qualitative data analysis.

Thematic analysis was conducted to code the deidentified interview transcripts. Thematic analysis allows the identification, analysis, description, and reporting of themes found in qualitative data [58]. We established the validity and reliability of the thematic analysis results by following the Lincoln and Guba [52] criteria for conducting qualitative research. (researcher triangulation, code reviews, expert feedback, and resolution meetings [52,59]).

First, we inductively coded (ie, created low-level codes) our interview transcripts without referring to our conceptual model (Figure 1). Inductive coding allowed us to capture phenomenological user experiences with flu@home. Second, after inductive coding, we referred to an a priori high-level coding schema (Textbox 2) reflective of our conceptual model (Figure 1). In particular, we reviewed our low-level codes to determine potential connections with high-level concepts (ie, engagement, enablement, empowerment, and activation). During this step, we found conceptual associations between low-level codes and high-level concepts in the model.

**Table 1:** Demographic data of the sample frame (phase 2 participants) used for semistructured interview (phase 3).

Phase (sample size)		Phase 2: trial of flu@home (n=724), n (%)	Phase 3: invited to participate in semistructured interviews (n=115), n (%)	Phase 3: completed semistructured interviews (n=31), n (%)
**Age (years)**				
	18-24	86 (11.9)	12 (10.4)	3 (9.7)
	25-34	204 (28.2)	34 (29.6)	6 (19.4)
	35-44	199 (27.5)	38 (33)	11 (35.5)
	45-64	188 (25.9)	21 (18.3)	8 (25.8)
	≥65	47 (6.5)	10 (8.7)	3 (9.7)
**Ethnicity**				
	White	510 (70.4)	78 (67.8)	21 (67.7)
	Black or African American	63 (8.7)	10 (8.7)	6 (19.4)
	Asian	60 (8.3)	8 (6.9)	0 (0)
	Native Hawaiian or other Pacific Islander	4 (0.6)	1 (0.9)	1 (3.2)
	American Indian or Alaska Native	17 (2.4)	18 (15.7)	1 (3.2)
	N/A^a^, other, or prefer not to say	70 (9.7)	2 (1.7)	2 (6.5)
**Geographic representation**				
	West	214 (29.6)	43 (37.4)	14 (45.2)
	Midwest	139 (19.2)	21 (18.3)	5 (16.1)
	Southwest	12 (1.7)	2 (1.7)	1 (3.2)
	Northeast	197 (27.2)	32 (27.8)	4 (12.9)
	Southeast	162 (22.4)	17 (14.8)	7 (22.6)

^a^N/A: not applicable.

**Categories of factors (engagement; enablement) and choices (empowerment and activation).**

**Engagement**

- Intrinsic influences
- Extrinsic influences

**Enablement**

- Considering the quality
- Assessing personal capacity

**Empowerment and activation**

- Patient-familiar
- Patient-distant
- Public-familiar
- Public-distant

To enhance research reliability and validity, the research team used a constant comparison method to refine coding [49,60]. This procedure involved 2 coders (unfamiliar with the conceptual model) and an internal auditor (a research team member with extensive qualitative research methods expertise who was familiar with the conceptual model). The internal auditor also reviewed the structure, syntax, and labeling of the final coding schema and performed a code review of 100% of the coded quotes to ensure alignment with the final coding structure. The coding team met regularly to iteratively discuss and reconcile initial inductive coding, which included ensuring that codes were supported by linked quotes, refining coding categories, and reviewing emerging themes. Once a detailed inductive coding scheme was in place, the coding team independently and collectively identified and reconciled the conceptual associations between the low-level codes (created because of inductive coding) and high-level concepts (Textbox 2). The team members traced the codes forward from code to model and backward from model to detailed codes and their underlying quotes from the transcripts. Throughout both inductive and deductive coding, the coding team collectively discussed and resolved any identified issues with codes associated with supporting quotes, as well as the structure, syntax, and labeling of the final comprehensive model. Reconciling points mainly focused on combining various subcodes and updating the labels and definitions of individual codes.

### Surveys

The survey contained 3 sections. The first section contained questions about the participants’ prior engagement with the smart HT for influenza. The second section contained questions about the impact of the COVID-19 pandemic on participants’ future engagement and enablement decisions associated with smart HT for influenza. Finally, the third section contained questions about participants’ potential engagement, enablement, empowerment, and activation decisions associated with smart HT for COVID-19. The survey did not ask participants to provide demographic information, given institutional review board cautions in asking demographics to preserve the anonymous nature of the survey (thus, we were unable to perform an analysis of demographic and categorical data).

Insights from semistructured interviews informed the survey questions, which were developed to validate and further explore factors affecting decision points associated with engagement and enablement, as well as choices resulting from empowerment and activation. Further exploration of factors was conducted because of the emergence of the COVID-19 pandemic. The research team included additional questions related to engagement, empowerment, and enablement associated with smart HT for COVID-19. The survey questions used ordinal and categorical response options. For ordinal questions, the research team used a 5-item Likert scale for responses ranging from strongly agree to strongly disagree. The Likert scale is an efficient and reliable technique for examining individual attitudes and perceptions [61,62]. Compared with 7- or 10-point scales, 5-point Likert scales have been shown to reduce survey fatigue and increase response rates [63,64]. In addition to Likert-type questions, the survey included a few categorical responses that aligned with the nature and purpose of the questions and were not well-suited to a Likert scale. The research team reviewed all the survey questions to ensure clarity. The results are shared in Multimedia Appendix 2, and the *Results* section of this paper showcases the survey questions relevant to this study.

Participants from phase 2 (trial of flu@home) were recruited to complete the survey (Table 1 shows participants’ demographics from phase 2). The participants received an initial recruitment email and a follow-up email a week later. They did not receive compensation for completing the survey, and no demographic information was collected.

The anonymous survey was administered using Qualtrics Survey Platform software [65] in June 2020. In total, 38.2% (282/739) of eligible individuals from phase 2 completed the survey.

Survey data were analyzed using descriptive statistics (mode, as well as response distribution counts and percentages), as appropriate for Likert scales [66].

### Ethics Approval

The study design was approved by the University of Washington Institutional Review Board (STUDY00007627).

## Results

### Smart HT Engagement Overview

We aligned the general structure of our results with the Smart HT–Empowered Activation Model (Figure 1), which showcases the emergent states of engagement, enablement, empowerment, and activation covered by this study. We provide associated key interviews and survey highlights under the associated Smart HT–Empowered Activation Model sections. We provide further details of our findings in Multimedia Appendix 3 (interview evidence trace table) and Multimedia Appendix 2 (details of ordinal survey response questions).

### Acquisition of Motivation: Smart HT Engagement

#### Overview

**Figure 3:**
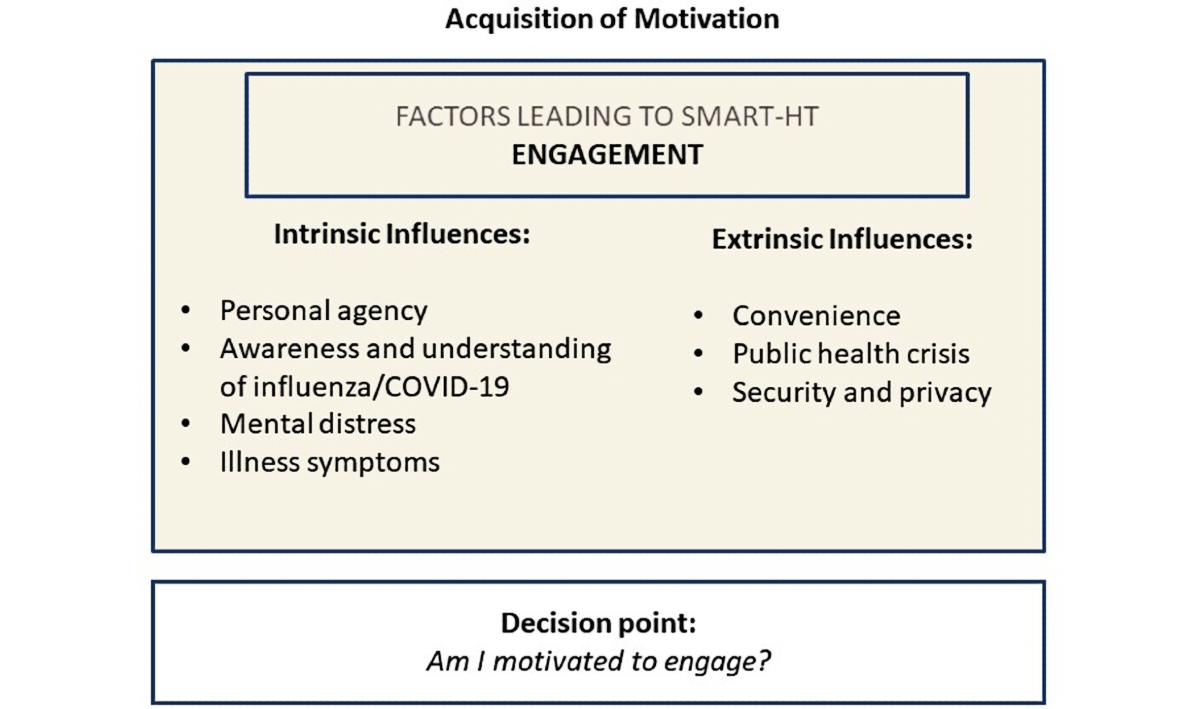
Factors affecting engagement. Smart HT: smartphone-supported home testing.

The acquisition of motivation involved both intrinsic and extrinsic influences. We identified intrinsic influences covering specific states (eg, mental distress) and traits (eg, personal agency) of the users. In addition, the identified extrinsic influences covered specific characteristics of smart HT (eg, convenience) and environmental conditions (eg, public health crisis). Figure 3 summarizes these findings.

#### Smart HT Engagement: Intrinsic Influence Factors

Intrinsic influences include personal agency, awareness and understanding of viral infection (influenza; COVID-19), mental distress, and illness symptoms.

*Personal agency* was stated as a contributing factor to engaging with smart HT. The participants generally expressed high personal agency in managing their health. Most interview participants articulated that they believed they were primarily responsible for managing their health or acted as equal partners in their care with their providers. Interviewees perceived the flu@home smart HT as providing them with choice and control over when and where to conduct diagnostic testing.

This technology appealed to interview participants who proactively managed their health, as well as individuals who self-identified as having poor health behaviors. Therefore, although we recognize that this may play a role in the decision process for some, there was no general consensus that *health behaviors and attitudes* were influential factors in participants’ considerations of engaging with smart HT.

*Awareness and understanding of the illness* (in this study, influenza and COVID-19) was a factor in the interview participants’ consideration of engaging with smart HT. Interviewees’ beliefs regarding the severity of seasonal influenza varied greatly. Those who believed influenza was a minor illness (frequently referencing a *cold*) were less motivated to engage with a self-test than those who perceived influenza as a serious health concern.

Regarding when health consumers might be motivated to perform a smart HT, survey responses indicated (Multimedia Appendix 2) that 96.5% (272/282) of participants strongly or somewhat agreed that they would use the flu@home test if they experienced ILI *symptoms*. Only 32.6% (92/282) of participants strongly agreed that they would use flu@home testing when asymptomatic. As for COVID-19 testing, 95.8% (249/260) of the participants strongly agreed or somewhat agreed that, if available, they would use a COVID-19 home test when experiencing COVID-19–related symptoms. Most survey respondents (196/260, 75.4% strongly agreed or somewhat agreed) stated that they would use a COVID-19 home test, even if they did not experience symptoms common to COVID-19. To further understand the relationship between the acquisition of information and motivation stages, it is notable that symptom onset did not necessarily correlate with the preferred timing of test acquisition. Specifically, some interview participants indicated that they might opt to proactively purchase smart HT to keep at home for convenient access when symptoms (and the need for testing) arise. One of the participants explained the following:

I should keep some of the kits at home on an ongoing basis so that anytime I feel I have this fever, sneezing, runny nose and all those symptoms.

Moreover, interview participants also indicated that mitigating *mental distress* at a time when they were also feeling physically unwell was a motivating factor for smart HT. When asked what would make them inclined to use a smart HT instead of going to a physician, one participant said the following:

And I think that it would be more convenient because sometimes you just don’t feel well and feel like leaving the house. It’d be nice because I feel like you might be able to find out results sooner than if you wait to go to the doctor...I feel like there’s less pressure when you’re at home, and you’re more relaxed...I shouldn’t say pressure, but less stress. There’s always some, at least for me, levels of extra stress going to the doctor just in general. Getting out of the house and sitting in the waiting room, and being back there, and just kind of like nervous and stuff, waiting to see what the doctor’s going say.

We further delved into this issue in the survey. Although testing at home when feeling ill may mitigate the stress of traveling and waiting to see a provider to perform a diagnostic test, a concern about the future of home testing is that test results indicating a serious health condition (such as COVID-19) delivered without provider support could cause mental distress. According to our survey results, 74.1% (192/259) of the survey respondents indicated that they would find testing positive (ie, learning that they contracted the COVID-19 illness) in the at-home context (smart HT) no more distressing than learning of their diagnosis in other health care settings (Multimedia Appendix 4).

#### Smart HT Engagement: Extrinsic Influence Factors

Extrinsic influences include convenience, public health crises, and security and privacy. Participants overwhelmingly shared that *convenience* was a primary motivating factor in considering smart HT use. For example, one of the participants stated the following:

Just the idea of being able to do home-based checking interests me. It sounds like it has promise to me. And I think that a lot of people might use something like that rather than going through the grief of trying to get a doctor’s appointment, which is hard to do here.

*Convenience* of engaging with smart HT manifested in avoiding the burden of visiting a provider in person. Participants mentioned some of the burdens of visiting a provider, including difficulty in scheduling appointments, finding appointments that would not require taking time off from work, and difficulty meeting a provider in person while caring for young children. Some interview participants indicated that they were particularly motivated to use smart HT to diagnose their children.

Furthermore, survey respondents indicated that a *public health crisis* (ie, the COVID-19 pandemic) affected their decision to engage with smart HT for viral infections. Participants’ attitudes toward smart HT were affected by the COVID-19 pandemic (Multimedia Appendix 2). For example, when asked, “Which of the following best describes how COVID-19 influences your thoughts about using flu@home to test for common, seasonal flu if you have symptoms?” the most common response (132/263, 50.2%) was, “I am much more likely to use flu@home for testing of common, seasonal flu.”

Interview participants also identified the *security and privacy* of the data generated from smart HT as a factor of engagement. Participants were most concerned that their health data would be sold or shared without their consent if tests were provided via web-based sources.

### Acquisition of Ability: Enablement Through Smart HT

**Figure 4:**
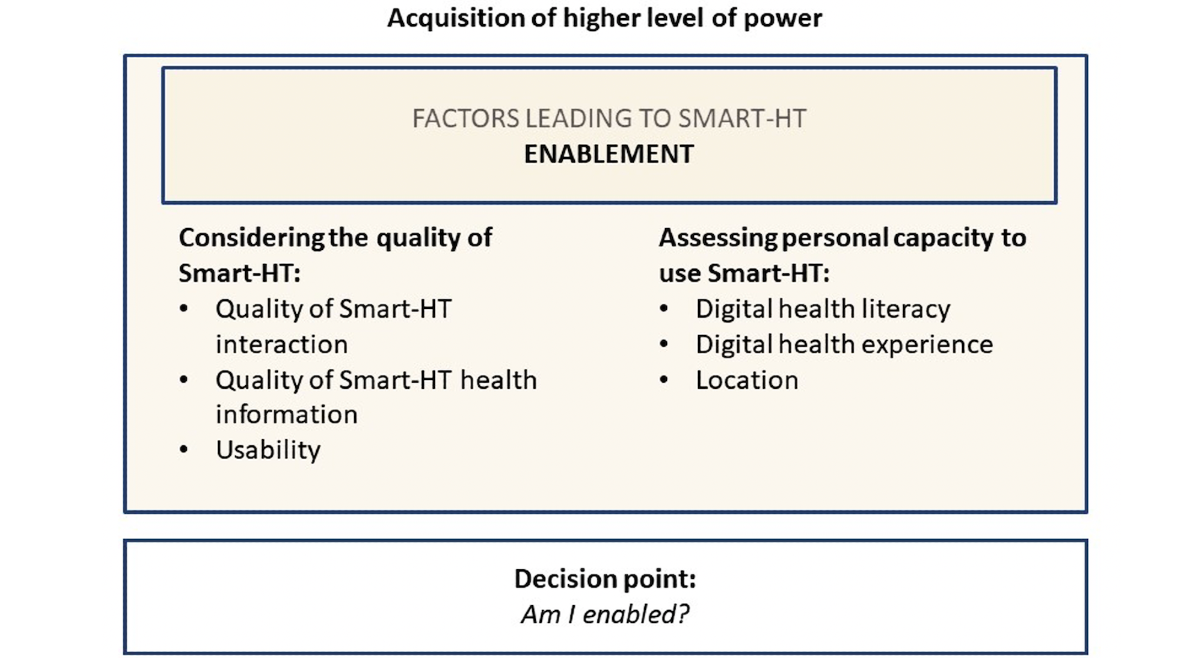
Factors affecting enablement. Smart HT: smartphone-supported home testing.

The interview data revealed various factors leading to participants’ enablement facilitated by smart HT. We categorized these factors into two groups: (1) considering the quality of smart HT and (2) assessing personality capacity to use smart HT (Figure 4).

#### Enablement: Considering the Quality of Smart HT

Regarding the *quality of smart HT interactions*, interview participants shared that they valued a digital experience that segmented the testing process into small, digestible, step-by-step instructions with illustrations and videos in the app. The participants also appreciated the built-in timers to reduce the likelihood of errors.

Interviewees also considered the *quality of smart HT health information.* Participants indicated that they understood and retained content that differentiated common cold and influenza symptoms, as well as information provided when it was appropriate to consult a provider. Participants were also receptive to the flu@home app, including general facts about influenza, such as how many people are affected each year, and other information to help remind consumers about the importance of preventive measures (such as vaccination).

Participants also shared that they would like future iterations of flu@home to include more health information, such as explaining “how contagious the flu is,” and recommendations for managing the illness. For example, one of the participants described the following:

I think one of the biggest things we do that we shouldn’t do in this day and age is just trying to take something [medication] to suppress the symptoms and then head right back to work or other things. I think having some statistics or data about the dangers of taking the flu out of the house...would be really helpful.

In evaluating the *usability* of the testing processes, interview participants described the flu@home system as easy to use, attributable to clearly labeled kit contents and simple nasal swabbing procedures with mild or no discomfort. These features seemed to influence their decision to perform the test. Incorporating a smartphone app into home testing broadens the potential features that test developers can incorporate into smart HT. The study team presented many potential features in interviews with participants to consider enhanced value. Added features that appealed to the participants included the ability to share their home test results with their providers. Many participants were interested in smart HT that incorporated data collected from a wearable device. In addition, participants were interested in receiving alerts if an RVI outbreak occurred in or near their community. Nearly all participants indicated that they would be willing to share deidentified data to contribute to their community's public health management of influenza. However, participants’ responses varied greatly regarding whether they would value gamification features in smart HT.

#### Enablement: Assessing Personal Capacity to Use Smart HT

The interview participants generally indicated that they felt capable of completing the smart HT test. Specifically, participants indicated that they felt enabled to complete the test with the digital guidance provided in the flu@home app, thus, informing their belief that they completed the swabbing procedures as instructed in the app and that they felt capable of completing the smart HT again in the future.

*Digital health literacy* was a critical factor in the assessment of personal capacity. All participants indicated that they could download the app, order the kit, and complete the testing without clinician supervision. They demonstrated that they had the skills to complete these actions because they completed the pilot study in phase 1. However, frequent *digital health experience* (eg, using mobile apps and wearables) was not a universal factor in participants’ assessment of their capacity to use smart HT. Although some participants described their lifestyle as including the use of a wearable device or health app, others said they had not found such products to be valuable and did not use digital health resources unless necessary for clinical care.

Moreover, the *location* of the testing was another critical factor in assessing the personal capacity to perform the test. Participants generally indicated that finding a specific place in their home was essential for performing the test. Participants mentioned various locations where they could perform the test (eg, bathroom, bedroom, and kitchen) and the specific characteristics of such locations. For instance, some participants indicated that it was critical for the location where they performed the test to be clean. In addition, one participant alluded to privacy as an essential aspect of choosing a location for the test. This participant said the following:

I feel like I would do it at home because there’s no other people around. They wouldn’t just see me stick something, the little test tube up my nose, or whatever. Can’t even think of what it’s called.

### Acquisition of Higher Level of Power: Empowerment and Activation

#### Empowerment and Activation Overview

In the case of smart HT, empowerment and activation involve 2 sequential points. The first factor was the intention to perform the test. The second was the intention to act on confirmation of influenza results from completing the smart HT test.

#### Empowerment and Activation: Intention to Test Using Smart HT

We found evidence that the study population was empowered to use smart HT to test for viruses. Approximately 81.5% (207/254) of the participants who completed the survey indicated that they would prefer to test for viruses at home rather than a test conducted by a health care provider (Multimedia Appendix 4). Moreover, survey results indicated that 94.6% (265/280) of participants somewhat to strongly agreed that they would use the smart HT test kit for influenza in the future, regardless of pandemic conditions (see Multimedia Appendix 2 for details). Analogously, interview participants shared that they intended to acquire and use smart HT in the future, once commercially available.

Survey responses showed that many people were willing to test for COVID-19 regularly, every 14 days (97/260, 37.3%), or monthly (93/260, 35.8%) to ensure that they were healthy and could interact with others (Multimedia Appendix 4).

Moreover, there were indications that an empowerment and activation process could have spillover effects on other possibilities. During the interviews, participants also mentioned various other (not necessarily viral) health conditions that they would be interested in using smart HT in the future: common cold, dementia, bronchitis, cancer, diabetes, pneumonia, sinus infections, hepatitis, and many others.

#### Empowerment and Activation: Intention to Act on Confirming Influenza Results

**Figure 5:**
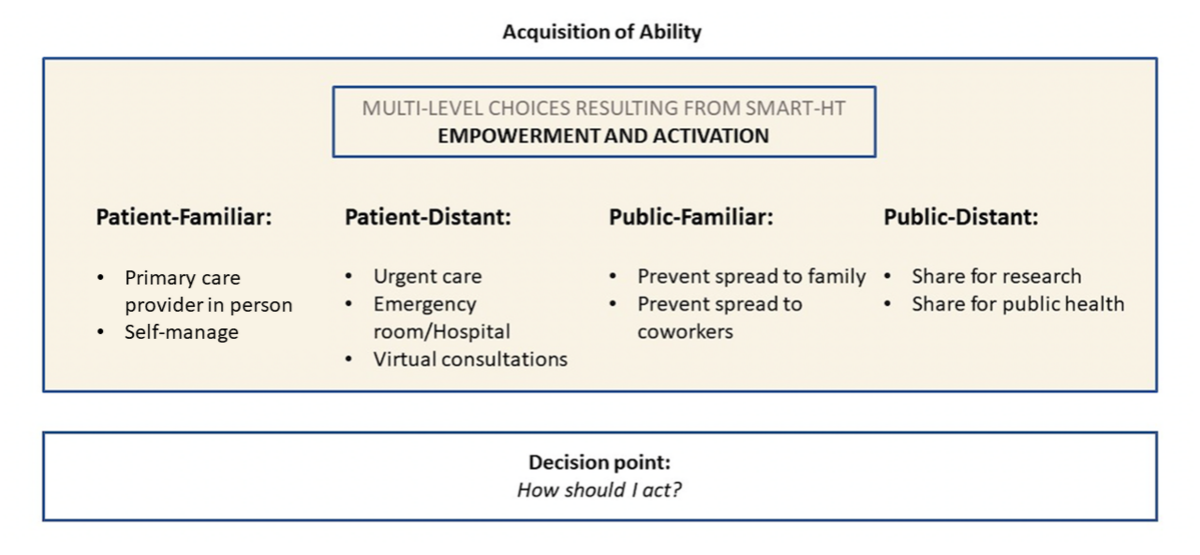
Multilevel choices resulting from empowerment and activation. Smart HT: smartphone-supported home testing.

The findings indicate that individuals consider actions related to all four themes regarding acting on positive smart HT results:(1) patient-familiar, (2) patient-distant, (3) public-familiar, and (4) public-distant. Figure 5 summarizes these findings.

Textbox 3 summarizes the qualitative results that support multilevel choices resulting from empowerment and activation.

In looking holistically at multilevel choices resulting from empowerment and activation, some interview participants gravitated toward *patient-familiar* means of self-care (eg, self-management or primary care provider appointment). However, other interview participants were open to less familiar forms of care, such as urgent care or emergency room visits or seeking a telemedicine (virtual) consultation *(patient-distant).* Limited access to care for reasons such as rural living status and insurance coverage were mentioned in the interviews as deciding factors for self-management of illness or high motivation for a virtual consultation. In addition, the rationale shared for virtual consultation included convenience and treatment expedience (eg, antiviral prescription), potentially minimizing the chance of spreading the illness and acquiring a new illness during a provider visit.

To assess whether participants’ attitudes toward telemedicine (virtual care) changed because of COVID-19, we asked them to reflect on their initial willingness to seek virtual care. Survey respondents indicated that they were equally willing to have a virtual care appointment (telemedicine) after testing positive for influenza and COVID-19. Approximately 93.9% (265/282) of the participants strongly or somewhat agreed that they would have been willing to have a virtual appointment if the flu@home results had returned positive. Similarly, 93.1% (242/260) of the participants somewhat or strongly agreed that they would have been willing to have a virtual appointment if their COVID-19 test results returned positive.

It is also noteworthy that interview participants reported that the responsibility of caring for young children influenced their test result response choices, with parents of young children sometimes opting for distance care for themselves but preferring in-person care for their children.

Regarding public considerations, interview participants indicated that they were receptive to contributing to the public health management of a viral outbreak (*public-familiar)*. In addition to the *public-familiar* means of managing the spread provided in Multimedia Appendix 4 for influenza, most survey respondents indicated that they were taking some of the recommended actions to prevent the spread of COVID-19.

Regarding the *public-distant* choice, participants indicated that they were willing to share data for research purposes. Although the contribution to research generally denotes a distal relationship, participants indicated that they were more likely to participate in research studies if they were familiar with the research organization (trusting the entity to secure their data and maintain confidentiality). Moreover, most participants indicated that the COVID-19 pandemic influenced their motivation to share their smart HT results anonymously for public surveillance or research purposes. The interview data also seemed to indicate an escalated motivation for parents to contribute to the community or public health management of influenza.

**Key findings from interviews related to multilevel choices resulting from empowerment and activation.**

**Patient-familiar**

- Self-manage: Reasons for self-management included assumptions that a provider would tell them to rest at home and take over-the-counter medication to manage their illness anyway and the cost of care (particularly for the uninsured). Motivations for moving from self-care included the perceived need for prescription medication.
- Primary care provider in person: Participants indicating that they would seek an appointment with their provider frequently referenced an established, trusting relationship with their primary care provider: some referenced pre-existing conditions that could create health care complexities with influenza.

**Patient-distant**

- Urgent care: Rationale for urgent care as a form of provider engagement included reference to accessibility, namely, urgent care clinic during weekend or evening hours.
- Hospital emergency room: Some participants indicated that they would seek care from the emergency room as their default option when unsure how to manage a health issue.
- Virtual consultation: Virtual consultation was referenced as a means of convenient verification of diagnosis and a quick means to obtain treatment (ie, prescriptions).

**Public-familiar**

- Prevent spread to family: Although participants referenced both quarantining in and sanitizing their homes to prevent spread to family, they also shared practical challenges, particularly with quarantine.
- Prevent spread to coworkers: Participants mentioned the preference to stay home when sick with influenza symptoms to prevent spread to coworkers and more distant relationships (eg, public transit commuters). There was also mention of practical challenges because of some work arrangements.

**Public—distant**

- Share for research: Some participants felt that anonymously sharing self-test results could contribute to improved influenza vaccine development.
- Share for public health: Participants were generally willing to share self-test results for surveillance if done anonymously. They also indicated that they would personally reference a local or neighborhood-level influenza map in making prevention choices.

## Discussion

### Principal Findings

Smart HT aspires to facilitate the success and impact of home-based diagnostic testing by coupling the diagnostic procedure with technological supports [12-14]. Our findings implicitly signal promising aspects of coupling home-based medical procedures with digital support. However, for smart HT to achieve its intended use advantages, health consumers must have increasing levels of awareness, motivation, and ability to perform home-based diagnostic tests and act on results appropriately. Our mixed methods study results provide insight into the nuanced and myriad factors that affect engagement and enablement with smart HT for viral infections, as well as how empowered users intend to respond to smart HT results. Overall, this study extends a stream of past work by exploring each of these concepts (patient engagement, empowerment, activation, and enablement) in the smart HT context [17]. Our final Smart HT–Empowered Activation Model may have implications for an increased understanding of engagement, enablement, and empowerment in other HIT contexts.

Essentially, we contribute to the existing knowledge by *opening the black box* of engagement, enablement, and empowerment by contextualizing these constructs in the context of smart HT for viral infections. We were guided by the Fumagalli Concept Map of Engagement and Neighboring Concepts [17] and the DIEGO [18]. These models are based on an extensive literature review that depicts a health consumer’s progression toward the acquisition of a higher level of power (g, appreciating one’s role in health care and managing one’s health) that allows them to directly participate in the care process. Our results identify factors (from the perspective of the health consumer) that come into play for the engagement, enablement, and empowerment emergent states and frame our findings in the Smart HT–Empowered Activation Model.

Our findings highlight the complexity of digital health engagement. One of the most apparent elements of complexity is the number of factors that come into play during the empowered activation journey. For smart HT information technology developers, this indicates the importance of having a strategy to consider, leverage, and support various factors that lead to successfully performing the test and acting on test results, essentially, a journey map (as noted in design thinking [67]) that showcases the potential of the technology to support the test process.

Upon further reviewing our model through the lens of complexity, it is noteworthy that various factors involving the acquisition of motivation (engagement) can change over time. For example, regarding intrinsic factors, changing illness symptoms and awareness and understanding of RVIs can affect motivation to engage with smart HT. This timing element is something to consider, particularly in the role of technology in message engagement considerations. Public health crises, an extrinsic example, can influence an individual’s motivation to use smart HT. Smart HT technology features can help health consumers evaluate the safety and feasibility of testing at home versus seeking testing by other means during public health crises. This motivating factor begets developers and perhaps policy makers to promote the use of smart HT with demonstrated efficacy during a public health crisis.

Regarding intrinsic motivation, smart HT technology can provide information to enhance a user’s awareness and understanding of a specific respiratory illness. Providing this educational information also introduces some assessment regarding how much information is provided, when to provide the information, and whether information needs to be tailored to a particular user’s capabilities, base knowledge, or interest in the information. Additionally, it is important to note that some factors of engagement may be more important than others. For example, there were mixed findings regarding the importance of participants’ general health behaviors and attitudes. Some participants indicated that they generally practiced healthy behaviors, whereas others indicated the opposite. This factor may be subordinate to other factors (eg, personal agency and illness symptoms). The relative importance of these factors should be explored in future research.

Furthermore, regarding the acquisition of motivation (engagement), we found evidence that individuals did not anticipate feeling more distress when learning that they have an RVI at home using smart HT than when learning about their health status in a clinical setting. However, research indicates that diagnosing different health conditions such as cancer, dementia, and COVID-19 can evoke emotional distress [68-70]. Limited research assessing the mental distress of home testing exists. As more home-based diagnostic tools are developed and available for consumer use for various health conditions, future research is needed to understand the mental distress of receiving different types of diagnoses through a home-based test compared with clinical settings. Anticipating and mitigating mental anguish because of a positive test result may be worth considering in the design and use of smart HT. Pretest and posttest counseling have been suggested in some forms of home testing [71]. When relevant, smart HT technology features and functions may either provide functions to mitigate distress directly or refer the user to resources for assistance in managing distress.

The study shows that in the case of contagious diseases, multiple level factors need to be considered to have a robust smart HT. The acquisition of a higher level of power (empowerment and activation) involves decisions at multiple levels, which can have both personal and far-reaching impacts. For example, informed individuals may vary in their patient, familiar, distant, and public actions when testing positive for a viral infection. Ideally, this choice variance is because of an informed decision process and not because of missing information or misinformation. Therefore, a key role of smart HT during the acquisition of ability (enablement) stage is to prepare the individual performing the test for the multiple downstream choices resulting from enablement. In response, developers may want to embed quality information and various paths of action into the design and functions of smart HT to support an informed empowerment and activation decision-making process. The multiple choices presented to a patient upon receipt of their positive test results should be carefully considered when developers design smart HTs to reduce choice complexity. It is particularly important to ensure that patients are not overwhelmed with too many choices, as too many options can impair an individual’s subsequent self-control (and, therefore, personal agency) [72]. Furthermore, choices should be limited to *good choices*, indicating choices that align with the overall purpose of smart HT. For instance, if a patient tests positive for influenza, smart HT can provide them with a set of suggestions on managing their conditions and preventing their spread to other people. In addition, there can be an option for digitally sharing the results for research or public health purposes with smart HT. Moreover, smart HT might provide easy access to a digital provider (telemedicine) for treatment. To further minimize complexity and guarantee choices that keep the smart HT user heading in the direction in which they want to go, developers should try to limit recommendations or choices to those tailored to individual smart HT users. In addition, to facilitate empowerment regarding tailored choices, smart HT should provide clear guidance regarding the next steps required and the use of information for each possible option.

In addition to providing a better understanding of the affirmative path of the Smart HT–Empowered Activation Model, our work provides a foundation for future work to explore other paths through the model. For example, future empirical research could explore the relative importance of the identified factors or whether the factors would hold in the context of other forms of smart HT. Regarding the latter, participants did mention potential interest in various forms of smart HT for both acute and chronic conditions. Future research could explore and validate the factors of engagement, empowerment, and enablement (derived in this study) in other contexts mentioned by the participants.

### Limitations

As with most studies that include qualitative methods, the generalization of the results must be approached with some caution. Although the study included a diverse set of participants in terms of age and geography, it had some limitations. Most notably, our study population included only participants in the flu@home study. Attitudes toward home testing may differ among individuals who did not experience this specific smart HT or type of respiratory testing. In addition, this study was conducted early during the COVID-19 pandemic. COVID-19 home testing and self-swabbing availability and experiences during the pandemic might have implications for smart HT for influenza and other home-based diagnostic testing. Overall, we strongly encourage future research to consider our findings in other smart HT and HIT contexts.

### Conclusions

Through our findings, we proposed and informed a Smart HT–Empowered Activation Model depicting an engagement, enablement, empowerment, and activation process for smart HT use. The resulting model underscores the need to understand and address the path to health consumer empowerment and activation for smart HT use, resulting in actions that provide maximum health benefits to individuals and society. Overall, this study provides a foundation for researchers and developers to explore and create successful engagement strategies to align with consumer digital health opportunities to promote prevention, self-care, and spread control of infectious viruses such as influenza.

## Appendix

Multimedia Appendix 1 Interview protocol.

Multimedia Appendix 2 Likert scale survey questions.

Multimedia Appendix 3 Evidence trace tables for analysis of interview data.

Multimedia Appendix 4 Survey results.

## Abbreviations

DIEGO: Digital Health Engagement Model
HIT: health information technology
ILI: influenza-like illness
RQ: research question
RVI: respiratory viral infection
smart HT: smartphone-supported home testing
