# Supplementary material for: Health Consumer Engagement, Enablement, and Empowerment in Smartphone-Enabled Home-Based Diagnostic Testing for Viral Infections: Mixed Methods Study
Source: JMIR Mhealth Uhealth. 2022 Jun 30;10(6):e34685. doi: 10.2196/34685 (PMC9284354; doi:10.2196/34685)
Supplement: Multimedia Appendix 1 [file mhealth_v10i6e34685_app1.docx]

Multimedia Appendix 1. Interview protocol.

FLU Study Interview (year 1 experience)

[**Aims 2**](#_heading=h.gjdgxs)

[**Model 2**](#_heading=h.30j0zll)

[Comprehensive model - all flu study efforts: 2](#_heading=h.1fob9te)

[This Study 3](#_heading=h.3znysh7)

[**Introduction 3**](#_heading=h.tyjcwt)

[Greeting and Description 3](#_heading=h.3dy6vkm)

[The Process 4](#_heading=h.1t3h5sf)

[Consent 4](#_heading=h.4d34og8)

[**Questions 4**](#_heading=h.2s8eyo1)

[Health Behaviors and Attitude Questions 5](#_heading=h.17dp8vu)

[Flu@home Experience 6](#_heading=h.3rdcrjn)

[Value/ Intention to Use in the Future 7](#_heading=h.lnxbz9)

[Activation 8](#_heading=h.35nkun2)

[Improvements and Extension *(only if time allows)* 9](#_heading=h.1ksv4uv)

[Final Catchall 10](#_heading=h.44sinio)

[**Closing 10**](#_heading=h.2jxsxqh)

[**References 10**](#_heading=h.z337ya)

# Aims

- Determine the “levels” of engagement various types of health consumers are willing to take an advanced role (testing and interacting with app) in their self-care, particularly with an acute illness, such as influenza-like illness (ILI), using technology.
- General usability assessment of the flu study system (i.e., app and home-based testing)

# Model

## Comprehensive model - all flu study efforts:


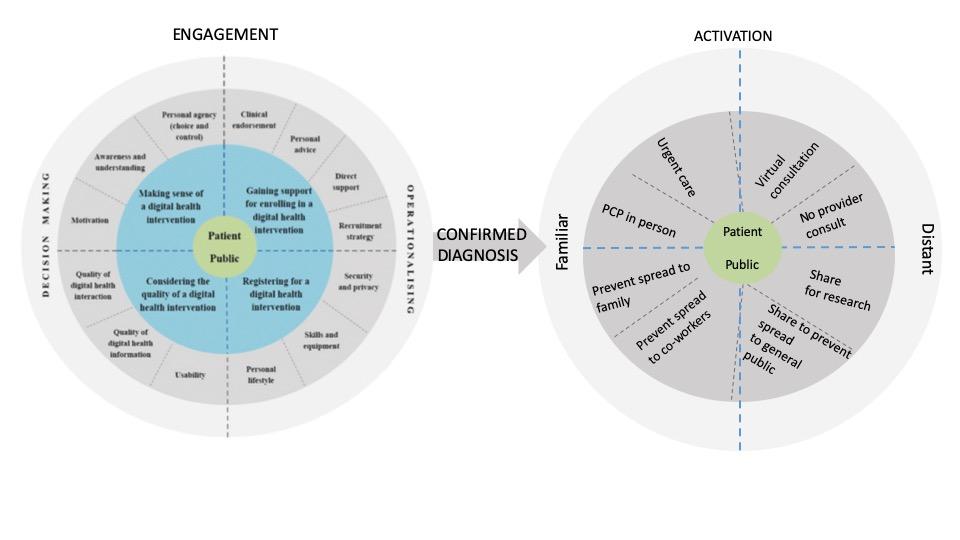


## This Study


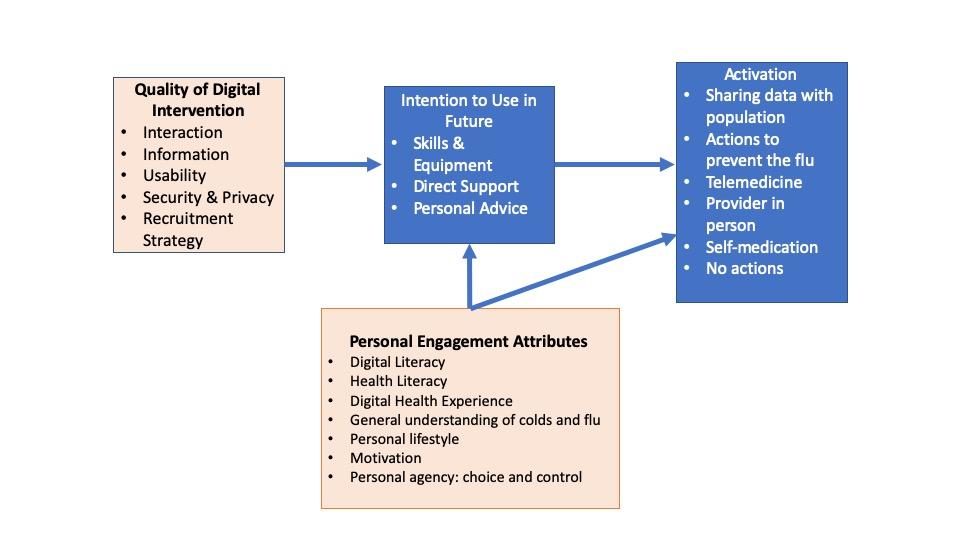


# Reminders

- Continue to probe whenever a respondent answers that something was “easy” to do/use. Ask why.
- Make sure conversation about comorbidities/ health conditions beyond the flu do not derail conversation.
- Make sure participants are available for 45 minutes.

# Introduction

## Greeting and Description

- Hello, my name is __________(Victoria or Cynthia) and I am a research scientist for the flu@home research study at the University of Washington Department of Family Medicine. I am joined by ___________(Cynthia, Polina, or Matthew), who is also part of the research team and will serve as an attentive listener to take notes and as some follow-up questions to our conversation based on what they hear.
- Can you hear us ok. Are you in a good location for this conversation.
- Is this still a good time for you. The interview will take approximately 45 minutes.
- Thank you for participating in this interview. We will talk about health care, health care technologies, and in particular flu@home.
- We would like to record the interview to accurately capture the information you provide. If you would like to go off recording at any time, just let us know and we can wait to discuss a question until after the recording is turned off. The transcripts will be kept confidentially and securely in our possession. The audio recordings will be destroyed after the transcriptions are verified.
- Participation in this study is voluntary. There will be no penalty if you decide not to continue at any point during the interview. Of course, we hope that you find this to be an interesting conversation and that you will be able to complete the discussion.

## The Process

- ______ will serve as the lead interviewer. ______ will ask you questions about your experience of participating in the flu study as well as relevant future implications of your participation.
- When you hear the term flu, we are referring to influenza. Do you have a preference for which word we use?
- We have grouped these questions into categories to facilitate the flow of the conversation.
- ________ will break for a moment at the end of each category to see if _____ has any follow-up questions or would like to clarify any comments made.

## Consent

Do you have any questions before we begin?

<If yes> Do you give us your permission to record our conversation?

We will now turn on a recorder to capture the interview.

# Questions

Key questions

## Health Behaviors and Attitude Questions

*First, we would like to ask you some general background questions about your general attitudes and behaviors regarding your health.*

1. What are some of the key things you do to take care of your health on a regular basis? Personal Lifestyle (O'Connor et al., 2016)
   1. *Do you visit doctors or dentists regularly, do annual physical?*
   2. *What do you usually do when you get sick (e.g. self-medicate, visit a doctor, etc.)?*
   3. *Do you exercise regularly and maintain good diet?*
2. How do you generally get information about your health? Health literacy (Chinn & McCarthy, 2013)
   1. Tell us a little about your experience with digital health such as healthcare web sites, on line groups, telemedicine, mHealth apps? Digital Health Experience (O'Connor et al., 2016)
      1. *Do you use them on a regular basis?*
      2. *Do you find them useful?*
3. *Do you every need assistance in interpreting the information from your providers? From other sources?*
   1. *Are you generally comfortable with providing information on forms or to your provider?*
4. What influences you to use technology for health and wellness purposes? Motivation (O'Connor et al., 2016)
   1. *Is there anything that would frustrate you about doing home based testing or interacting with an mHealth app?*
   2. *What can get in the way of motivation?*
5. So, now that we have talked about some of the things you do to take care of yourself can you provide me with a general overview regarding your thoughts on - *Do you believe that you are the person who is responsible for managing your health condition?*
   1. *Do you feel you are an equal partner with your provider in managing your health?*
   2. *In considering your response to your responsibility and partnership with your provider, in general what would you say a patients’ role is in managing their health?* Personal agency: choice and control (O'Connor et al., 2016)
6. Now let’s move to focusing on flu. In your opinion, how serious the flu? General understanding of colds and flu (O'Connor et al., 2016)
   1. *Do you think that flu require immediate care?*
   2. *Do you believe that a cold is just as serious as the flu.*
   3. *Do you believe that flu is a population health issue?*
   4. *How much of an inconvenience do colds and flu cause to someone who is ill?*
   5. *How much of an inconvenience do these illnesses cause to others such as family members, or people in the community around you?*

## Flu@home Experience

*We would now like to hear your overall experience with flu@home.*

1. What motivated you to be open to home-based flu testing? Motivation (O'Connor et al., 2016)
   1. *Is that what motivated you to be a part of the study?*
   2. *Did you think about stopping your participation in this study or doing the testing? Why?*
2. What do you think about the way that we found you and asked you to take part in this study (e.g. the website)? Was it effective in your opinion? Recruitment Strategy (O'Connor et al., 2016)
   1. *Are there any other alternative recruitment strategies that you believe would be more effective for home-based testing?*
   2. *Did you refer anyone to participate in the flu@home research study?*
      1. *How did you share it with them? (Website link, verbally/word of mouth, shared facebook link, shared link directly to app store)*
   3. *Did the fact that you could participate in this study from the convenience of your home affect your decision to participate?*
3. flu@home included multiple privacy and security safeguards. What did you look for to help ensure you that there was privacy and security? Security and Privacy (O'Connor et al., 2016)
   1. Do you have any privacy and security concerns about participating in home-based testing for the flu?
4. What are your general thoughts on features and design of the application? Usability (Ben-Zeev et al., 2014)
5. *Was there anything complicated about using the app?*
6. *What did you like the most about the app?*
7. *What did you like the least about the app?*
8. *What did you think about the navigation?*
9. *What did you think about design (e.g. icons, menus, and colors)?*
10. *Why was it easy to use?*
11. What do you think of the health related content provided in the app related to the colds and flu? Quality of digital health information (O'Connor et al., 2016)
12. *What would you have liked to have seen?*
13. *Was the content useful for you?*
14. *Did you learn anything from the content in the app?*
15. *Was it easy to follow and understand the content on colds and flu generated in the app? Or was the content too complex and medical to understand?*
16. *Do you think you became more aware about the flu and its impact on the population? Skills and equipment (O'Connor et al., 2016)*
17. *Is there any information about colds and flu that you think would be useful to add to the app (e.g. helping patients to decide on the future course of actions, treatment options, etc.)?*
18. How easy was the flu@home the actual home-based testing process? Usability (Ben-Zeev et al., 2014)
19. *Was there anything complicated about the process? Usability (Ben-Zeev et al., 2014)*
20. *Did you need any help from anyone at home when you did the flu test?*
21. *Do you think the time required to do the test was well spent?*
22. *Did it take too much time?*
23. *Did you have any difficulties with the nasal swab? Was it uncomfortable or painful?*
24. *Why was it easy to use?*
25. Were you successful in finding a place to take the test that was free of disruption?
    1. *Where did you complete the test? (Kitchen, living room, bedroom, bathroom, other?)*
26. How was the return mail process?
    1. *How did you mail back? (your mailbox, blue box…)*

## Value/ Intention to Use in the Future

*Since the testing allows you to know whether or not you have the flu, we would like to know if the results would have any implications for your future actions in relation to spreading the flu.*

1. Do you feel that if flu@home were generally available that you would have a good understanding of when to use flu@home in the future for you and family members? Personal agency: choice and control (O'Connor et al., 2016) Health literacy (Chinn & McCarthy, 2013)
2. Would you be open to using home-based testing for flu in the future, particularly, if immediate results were available? Motivation (O'Connor et al., 2016) (UTAUT – Intention to use)
   1. *Would you only be open in certain situations, such as:*
      1. *While traveling or other situation where you may be unable to visit your doctor*
      2. *Only when you reach a certain level of sickness*
      3. *If you didn’t have a friend/family member to drive you to a clinic*
   2. *Do you think having flu@home available to you to order from the Internet or from pick up from a pharmacy/drugstore would be valuable?*
3. *Would you promote flu@home use to your family and friends?*
4. How important is it for you that health providers have access to your personal test results? Direct Support (O'Connor et al., 2016); Personal Lifestyle (O'Connor et al., 2016)
   1. *Would you talk with your provider about the results?*
5. Do you think your participation in home-based flu testing could contribute to public health management of flu? Why? Personal agency: choice and control (O'Connor et al., 2016)
   1. For national tracking
   2. For community prevention

## Activation

1. If your results were positive for the flu, what would you do as next steps to manage your recovery? Activation (Hibbard et al., 2004)
2. *Would you go to an urgent care? no*
3. *Would you be open to a virtual consultation with a provider (virtual urgent care)?*
4. *Would you visit your primary care provider?*
5. *Would you self-medicate?*
6. *Would you do nothing? What if your symptoms got worse?*
7. What actions are you willing to take to prevent spread of the flu? Activation: Actions to prevent the flu (Hibbard et al., 2004)
   1. *to family?*
   2. *to co-workers?*
   3. *to general public?*
8. Under what conditions would you be willing to share your anonymous testing results to a cloud-based system to help map the spread of flu to benefit population health? Activation: Sharing data with population (Bartlett et al., 2018)
   1. Let’s consider this question from various aspects.
      1. *Assurance of privacy and anonymity?*
      2. *Certification that the data are used only for public health aggregation?*
      3. *High levels of trust in the intermediary requesting to use the data?*
      4. *Financial compensation?*
      5. *An opt-out option?*
      6. *Public health emergency?*
9. Would you be interested in viewing the anonymous flu map before you get sick? How would you use this tool?
   1. *When would you look at it?*
   2. *How often would you check it?*
   3. *Prefer to view map on phone or computer?*

## Improvements and Extension

1. What additional functionalities would you like to have in the app? I am going to provide a list of items and let me know if these things would be beneficial. Usability (Ben-Zeev et al., 2014)
2. *Would you be interested in keeping test results in order to have a history of when you had cold/flu?*
3. *Would you be interested in recording symptoms as to have a better understanding of flu vs. cold symptoms?*
4. *Would you be interested in recording treatments next step during this episode in order to understand what helps and what does not help?*
5. *mHealth gaming is gaining interest these days. What would you think if the testing or app contained more game-like features? What kind of game-like features would you prefer? Usability (Ben-Zeev et al., 2014).*
6. *Would you be willing to receive follow up notifications on your phone or by email after completing the flu@home test?*
   1. *Would you be willing to receive notifications before you got sick? (such as an alert indicating that flu is present in your community)*
7. What other types of home-based testing do you wish was available and why? Motivation (O'Connor et al., 2016)
8. If the following was required for home-based testing, would you be willing to do that?

i. Blood sample

ii. Urine sample

iii. Wearable health device (e.g. using a device like something to attach to your phone and listen to your heart)

1. If the flu or other forms of home-based testing become more mainstream, who or what would most influence you to do home-based testing? Direct Support (O'Connor et al., 2016)
   1. *Would/did you participate because of recommendations from your family/friends?*
   2. *Would/did you participate if the home-based flu testing was approved by your provider?*
   3. *Would/did you participate if the home-based flu testing was endorsed by health professionals and trusted organizations not affiliated with the organization providing the text?*
2. What is your reaction to the statement that home-based testing provides you with greater control over your healthcare? Personal agency: choice and control (O'Connor et al., 2016)
   1. Is it really important that you can set the time and location to do the testing?
   2. *To provide examples, woman can go into a drugstore to pick up a pregnancy test at one end and at the other end of the spectrum some tests require a scheduled appointment and specific location (some urine tests sometimes done in provider offices.*

## Final Catchall

Is there anything we missed that we should know in order to really understand your experience in this flu study and thoughts about home-based testing ?

Do you have any final recommendations for how we can make it easier and more engaging for people to participate in research studies of future versions of flu@home?

# Closing

Thank you for your participation. This interview has been informative and enjoyable. Please, don’t hesitate to reach out to us in case if you have any questions. Our email address is fluhelp@uw.edu.

# References

Bartlett, G., Macgibbon, B., Rubinowicz, A., Nease, C., Dawes, M., & Tamblyn, R. (2018). The Importance of Relevance: Willingness to Share eHealth Data for Family Medicine Research. In (Vol. 6).

Ben-Zeev, D., Brenner, C. J., Begale, M., Duffecy, J., Mohr, D. C., & Mueser, K. T. (2014). Feasibility, Acceptability, and Preliminary Efficacy of a Smartphone Intervention for Schizophrenia. In (Vol. 40, pp. 1244-1253).

Hibbard, J. H., Stockard, J., Mahoney, E. R., & Tusler, M. (2004). Development of the Patient Activation Measure (PAM): conceptualizing and measuring activation in patients and consumers. *Health services research*, *39*(4 Pt 1), 1005–1026. doi:10.1111/j.1475-6773.2004.00269.x

Chinn, D., & McCarthy, C. (2013). All Aspects of Health Literacy Scale (AAHLS): Developing a tool to measure functional, communicative and critical health literacy in primary healthcare settings. *Patient Education and Counseling, 90*(2), 247-253. doi:10.1016/j.pec.2012.10.019

O'Connor, S., Hanlon, P., O'Donnell, C. A., Garcia, S., Glanville, J., & Mair, F. S. (2016). Understanding factors affecting patient and public engagement and recruitment to digital health interventions: a systematic review of qualitative studies. *BMC medical informatics and decision making*, *16*(1), 120. doi:10.1186/s12911-016-0359-3

Venkatesh, Viswanath; Morris, Michael G.; Davis, Gordon B.; Davis, Fred D. (2003-01-01). "User Acceptance of Information Technology: Toward a Unified View". MIS Quarterly. **27** (3): 425–478. [*JSTOR*](https://en.wikipedia.org/wiki/JSTOR) [*30036540*](https://www.jstor.org/stable/30036540).

## 
