# Supplementary material for: Health Consumer Engagement, Enablement, and Empowerment in Smartphone-Enabled Home-Based Diagnostic Testing for Viral Infections: Mixed Methods Study
Source: JMIR Mhealth Uhealth. 2022 Jun 30;10(6):e34685. doi: 10.2196/34685 (PMC9284354; doi:10.2196/34685)
Supplement: Multimedia Appendix 2 [file mhealth_v10i6e34685_app2.docx]

# Multimedia Appendix 2. Likert Scale Survey Questions

### Survey Questions Related to Engagement Decision (Acquisition of Motivation)

| **Survey Question Stems and Responses – Engagement Decision** | Strongly  Agree | Somewhat Agree | Neither Agree or Disagree | Somewhat Disagree | Strongly Disagree | Total |
| --- | --- | --- | --- | --- | --- | --- |
| My initial reaction to flu@home was that I would be very likely to use flu@home test kit during flu season if I did experience symptoms. | 213 *** *(75.5%)* | 59  *(20.1%)* | 5  *(1.8%)* | 2  *(0.7%)* | 3  *(1.1%)* | 282  *(100.0%)* |
| My initial reaction to flu@home was that I would be very likely to use flu@home test kit during flu season if I did not have symptoms | 92 ***  *(32.6%)* | 55  *(19.5%)* | 43  *(15.3%)* | 52  *(18.4%)* | 40  *(14.2%)* | 282  *(100.0%)* |
| In general, I would use flu@home in the future, if available, to test for common, seasonal flu, even if there is no flu pandemic. | 195 *** *(69.6%)* | 70  *(25.0%)* | 8  *(2.9%)* | 5  *(1.8%)* | 2  *(0.7%)* | 280  *(100.0%)* |
| If available, I would use a COVID-19 home-test, if I experience symptoms common to COVID. | 227 *** *(87.3%)* | 22  *(8.5%)* | 5  *(1.9%)* | 0 | 6  *(2.3%)* | 260  *(100.0%)* |
| If available, I would use a COVID-19 home-test, even if I did not experience symptoms common to COVID. | 141 *** *(54.2%)* | 55  *(21.2%)* | 31  *(22.9%)* | 16  *(6.2%)* | 17  *(6.5%)* | 260  *(100.0%)* |
| In general, I would use flu@home in the future, if available, to test for common, seasonal flu, even if there is no flu pandemic. | 195 *** *(69.6%)* | 70  *(25.0%)* | 8  *(2.9%)* | 5  *(1.8%)* | 2  *(0.7%)* | 280  *(100.0%)* |
| If available, I would use a COVID-19 home-test, if I experience symptoms common to COVID. | 227 *** *(87.3%)* | 22  *(8.5%)* | 5  *(1.9%)* | 0 | 6  *(2.3%)* | 260  *(100.0%)* |
| If available, I would use a COVID-19 home-test, even if I did not experience symptoms common to COVID. | 141 *** *(54.3%)* | 55  *(21.2%)* | 31  *(22.9%)* | 16  *(6.2%)* | 17  *(6.5%)* | 260  *(100.0%)* |
|  | Much less likely | Somewhat less likely | Neutral | Somewhat more likely | Much more likely | Total |
| Which of the following best describes how COVID-19 influences your general thoughts about common, seasonal flu? | 24  *(9.1%)* | 21  *(11.8%)* | 116 ***  *(44.1%)* | 40  *(15.2%)* | 52  *(19.8%)* | 263  *(100.0%)* |
| Which of the following best describes how COVID-19 influences your thoughts about using flu@home to test for common, seasonal flu if you have symptoms? | 11  *(4.2%)* | 10  *(3.8%)* | 61  *(23.2%)* | 49  *(18.6%)* | 132  *(50.2%)**** | 263  *(100.0%)* |

Note: *** designates mode.

## Survey Results Related to Enablement Decision (Acquisition of Ability)

| **Patient - Distant** |  |  |  |  |  |  |
| --- | --- | --- | --- | --- | --- | --- |
| **Survey Question Stems and Responses – Empowerment and Activation Decision** | Strongly  Agree | Somewhat Agree | Neither Agree or Disagree | Somewhat Disagree | Strongly Disagree | Total |
| In thinking back on my experience, I would have been willing to have a virtual (telephone or video) call with a health care provider if my flu@home test results were positive for common, seasonal flu. | 217***  *(77.0%)* | 48  *(17.0%)* | 11  *(3.9%)* | 5  *(1.8%)* | 1  *(0.4%)* | 282  *(100.0%)* |
| I would be willing to have a virtual (telephone or video) call with a health care provider if my COVID-19 home-test results were positive. | 204***  *(78.5%)* | 38  *(14.6%)* | 9  *(3.5%)* | 4  *(1.5%)* | 5  *(1.9%)* | 260  (*100.0%)* |
| **Survey Question Stems and Responses – Enablement Decision** | Much less concerned | Somewhat less concerned | Neutral | Somewhat more concerned | Much more concerned | Total |
| Which of the following best describes how COVID-19 influences your thoughts about having a virtual (telephone or video) call with a health care provider if your flu@home test results were positive for common, seasonal flu? | 9  *(3.4%)* | 11  *(4.2%)* | 59  *(22.4%)* | 52  *(19.8%)* | 132***  *(50.2%)* | 263  *(100.0%)* |
| **Public- Distant** |  |  |  |  |  |  |
| **Survey Question Stems and Responses – Enablement Decision** | Strongly  Agree | Somewhat Agree | Neither Agree or Disagree | Somewhat Disagree | Strongly Disagree | Total |
| I would be willing to share my anonymous COVID-19 home-test results for public surveillance. | 198***  *(76.5%)* | 38  *(14.7%)* | 11  *(4.3%)* | 3  *(1.2%)* | 9  *(3.5%)* | 259  *(100.0%)* |
| I would be willing to share my anonymous COVID-19 home-test results for research purposes. | 216***  *(83.1%)* | 30  *(11.5%)* | 9  *(3.5%)* | 4  *(1.5%)* | 5  *(1.9%)* | 260  *(100.0%)* |
|  | Much less likely | Somewhat less likely | Neutral | Somewhat more likely | Much more likely | Total |
| Which of the following best describes how COVID-19 influences your thoughts about sharing your anonymous flu@home test results for public surveillance (tracking of flu)? | 10  *(3.8%)* | 12  *(4.6%)* | 61  *(23.2%)* | 47  *(17.9%)* | 133*** *(50.6%)* | 263  *(100.0%)* |
| Which of the following best describes how COVID-19 influences your thoughts about sharing your anonymous flu@home test results for research purposes? | 6  *(2.3%)* | 8  *(3.1%)* | 59  *(21.6%)* | 45  *(17.2%)* | 143*** *(50.8%)* | 261  *(100.0%)* |

Note: *** designates mode
