# Supplementary material for: Health Consumer Engagement, Enablement, and Empowerment in Smartphone-Enabled Home-Based Diagnostic Testing for Viral Infections: Mixed Methods Study
Source: JMIR Mhealth Uhealth. 2022 Jun 30;10(6):e34685. doi: 10.2196/34685 (PMC9284354; doi:10.2196/34685)
Supplement: Multimedia Appendix 3 [file mhealth_v10i6e34685_app3.docx]

## Multimedia Appendix 3 – Evidence Trace Tables for Analysis of Interview Data

| **Research model theme** | **Engagement Decision: Subtheme** | **Representative Quote** |
| --- | --- | --- |
| Intrinsic Influence | *Personal Agency* | *If you had it available at your home or your office or wherever you could do it, I think you'd be in a better situation to stay on top of things and be in better control.* |
|  | *Awareness and Understanding of influenza/COVID-19* | *It can be serious, and it can be very common. And it can be just something that looks like a really bad cold, or it can be something that has more serious side effects. And it also can be something that someone dies from. So, it can just be your every day I don't feel well with the fever, maybe vomiting, maybe not. Maybe a cough. Or it could be so severe that you're dehydrated, and you end up losing your life from it.* |
|  | *Health behaviors and attitudes* | *I actually have done somewhat of research on this a little. Because like I said, I had flu. So I had to educate my mind on how I can get better and get over having a flu. I learned drinking plenty of water actually helps your chances of not getting the flu. I also learned, well, my dad, he taught me this. He's old-fashioned; drinking hot tea and lemon and honey actually help you fight it off real fast too.*  *Frankly, I was not much worried about flu a couple of years ago. I keep thinking this is better that I get a flu shot and everything, never paid much attention, I would say five, six years ago. In the last few years, I have seen a lot of news, a lot of media attention or the flu is also sometimes not curable, so it may lead to that flu in some cases. That is basically more of the awareness now, so I make sure that I get the flu shot every year without any gaps. I follow for myself as well as for my family.* |
|  | *Mental distress* | *And I think that it would be more convenient because sometimes you just don't feel well and feel like leaving the house. …I feel like there's less pressure when you're at home, and you're more relaxed...I shouldn't say pressure, but less stress. There's always like some, at least for me, like levels of extra stress going to the doctor just in general. Getting out of the house and sitting in the waiting room, and being back there, and just kind of like nervous and stuff, waiting to see what the doctor's going say.* |
|  | *Illness symptoms* | *I should keep some of the kits at home on an ongoing basis so that anytime I feel I have this fever, sneezing, runny nose and all those symptoms*. |
| Extrinsic Influence | *Convenience* | *Just the idea of being able to do home-based checking interests me. And I think that a lot of people might use something like that rather than going through the grief of trying to get a doctor's appointment, which is hard to do here.* |
|  | *Security and Privacy* | *I did some research on your company prior to doing the tests and things doing the tests. So, I wanted to make sure it was a trustworthy organization and things of that nature.* |

| **Research model theme** | **Enablement Decision: Subtheme** | **Representative Quote** |
| --- | --- | --- |
| Considering the quality of a Smart-HT | *Quality of Smart-HT interaction* | *I thought that it was really smart that each step was a whole screen and then you had to press next to go to the next step instead of trying to fit multiple steps at once. It made it just a little easier. It could be easy for me, if there were multiple steps listed, to just kind of skim them, and I might've missed something. So, I thought it was really smart that each step was its own screen, and you have to physically move to the next screen before you saw the next step, so you had to read everything.* |
|  | *Quality of Smart-HT health information* | *If I remember correctly, it had facts and stuff that were popping up saying how many people were affected by the flu, and how many people were vaccinated, and what the flu symptoms actually were and when you should be worried, and all that stuff.... I think it wasn't overwhelming with the amount of information. It was also interesting, so that was nice. It was just the right amount to make it enjoyable to look at without being like, oh, my gosh, not more information.* |
|  | *Usability* | *The design of the box itself was really nice. And it all kind of folds up neatly inside there. It's got your little packaging clearly which ones you do in which order. What stuff you can throw away and what stuff you need to keep was in the app. It was telling you that along the way, too.* |
| Assessing personal capacity to use Smart- HT | *Digital health use* | *I actually have downloaded a few apps, but I didn't keep them for long just because I'm not really on my phone much. I'm in school half the time, so I barely even have time to be on it...Well, I had the ones for my asthma, and then I had ones that made sure I kept up with my health in general, keeping track of my body temperatures and blood pressures and stuff like that just because I do take asthmatic medication, so it makes me off limits with my heart rate and blood pressure and everything so I just try to keep on track. And ... I found an app that tracked when I was supposed to take my medication because sometimes, I just forget. So, this was like that one that was really, really helpful.* |
|  | *Digital health literacy* | Interviewer:  *Okay. When you talk about that website with the videos, did you contact a doctor through video before?*  Participant:  *Yes, I actually did. That was when I was on a medication for the flu. I actually had this flu. They had prescribed me a medicine, and it was making me shake. It would give me the shakes. I don't know why, but I felt like they needed to know, or I needed to know what I needed to do about this. So I reached out. The doctor, well the nurse, told me to make an appointment with my normal doctor and tell my doctor about that because that was a reaction to a medication.* |
|  | *Location* | *I feel like I would do it at home because there's no other people around. They wouldn't just see me stick something, the little test tube up my nose or whatever. Can't even think of what it's called.* |

| **Research model theme** | **Empowerment and Activation Decision: Subtheme** | **Representative Quotes Regarding Actions (particularly, if test positive)** |
| --- | --- | --- |
| Patient- Familiar | Self-manage | *And then for me personally, just because I'm an otherwise healthy adult, I would treat the symptoms and try to get more sleep and eat some chicken noodle soup. But I wouldn't, I don't think, need any special medical treatment or anything.* |
|  | Primary Care Provider (PCP) in-person visit | *I would go to my doctor. I've never had the flu and tried to tough it out without medication from the doctor because the medicines that they give me it's like... It's a prescription, there's nothing over the counter. So, me personally, I've never heard of over-the-counter flu medicine. It might be some, but I've never tried it.* |
| *Patient- Distant* | Emergency Room/Hospital Visit | *I'd probably just go to the emergency room. I usually go to the emergency room every time there's something wrong, and I'm not sure what it is.* |
|  | Urgent Care visit | *I don't have insurance currently, so I can't afford a primary care physician, but when I do have insurance I'm completely okay with having one of those. It's just a lot harder to get into them. For my daughter, I try to get her into it depending on what urgent care says. But urgent care is usually an easier option. It's more readily available. They have later hours; they have weekend hours, and it works better for our schedule. But she sees her pediatrician on her schedule, whatever the schedule is for toddlers. And then when I did have a doctor, I had a yearly checkup, and I'd go for certain issues. It’s just urgent care has always been an easier, more readily available option.* |
|  | Virtual consultation | *I would prefer [virtual appointment] because it's not like a very unique kind of illness you really have to go and talk to your doctor. The doctor knows about the flu, how to cure that, possibly some changes based on your lifestyle, your age, your gender and everything, and a telemedicine discussion with the doctor would be equally helpful and everything, and cost saving as well.* |
| Public - Familiar | Prevent spread to family and friends | *Just be clean. Just clean your surroundings. If you're at home and you know you're sick and you have people coming over, disinfect everything, use disinfectant spray to clean the house. Everything's tidy and clean, and if you're in public, wash your hands or wipe down where you were sitting just because you never know.* |
|  | Prevent spread to co-workers | *Call in sick to work. If I knew that I had the flu, I would take immediate action to try to not spread it around my community. And I would also, the next step that comes to mind is I would want to test the other people in my household. So, I'd want to test my baby, I'd want to test my husband to make sure that they also cannot spread it further in the community if they were to be positive…I would just try to stay home and not spread it further.* |
| *Public - Distant* | Share for research | *I guess if the research that you're doing helps make it and that you find out that it's a valid test, and then more people actually go, and they pick up these kits, and they can find out if they have the flu. Then if they know they have the flu, maybe they wouldn't go to work, so they'd stay home and get better before they're going in and exposing more people at work or going to school or things like that. So, they may just think their child has a cough for the last week, and they've been sending them to school, and they actually had flu. So, I think anything that starts with research can turn into something better that helps in the long run.* |
|  | Share for public health | *I would be pretty open to it [sharing for public health purposes] because if it's going to help somebody else prevent of getting the flu, I'm open to it. Because I know how it feels to have the flu and be miserable and not know any type of information. So, if it helps the next person with knowledge of the flu and how to get over it and how to get better, I'm all for it.* |
