# Supplementary material for: Health Consumer Engagement, Enablement, and Empowerment in Smartphone-Enabled Home-Based Diagnostic Testing for Viral Infections: Mixed Methods Study
Source: JMIR Mhealth Uhealth. 2022 Jun 30;10(6):e34685. doi: 10.2196/34685 (PMC9284354; doi:10.2196/34685)
Supplement: Multimedia Appendix 4 [file mhealth_v10i6e34685_app4.docx]

**Multimedia Appendix 4. Supporting Survey Results Graphs**

**Settings to obtain results**

**Modes of Testing**

**Frequency of testing**

**COVID-19 spread actions (participants directed to check all that apply)**
